# Comparability of automated human induced pluripotent stem cell culture: a pilot study

**DOI:** 10.1007/s00449-016-1659-9

**Published:** 2016-08-08

**Authors:** Peter R. T. Archibald, Amit Chandra, Dave Thomas, Olivier Chose, Emmanuelle Massouridès, Yacine Laâbi, David J. Williams

**Affiliations:** 1 Centre for Biological Engineering, Loughborough University, Loughborough, LE11 3TU UK; 2 TAP Biosystems, Part of the Sartorius Stedim Biotech Group, Royston, UK; 3 CECS/I-Stem, AFM Institute for Stem Cell Therapy and Exploration of Monogenic Diseases, 2 rue Henri Desbruères, 91100 Corbeil-Essonnes, France; 4 Cell and Gene Therapy Platform CMC, GlaxoSmithKline PLC, Stevenage, UK

**Keywords:** Automation, Centrifugation, Pluripotent stem cell, Characterisation, Scalable, Comparability

## Abstract

Consistent and robust manufacturing is essential for the translation of cell therapies, and the utilisation automation throughout the manufacturing process may allow for improvements in quality control, scalability, reproducibility and economics of the process. The aim of this study was to measure and establish the comparability between alternative process steps for the culture of hiPSCs. Consequently, the effects of manual centrifugation and automated non-centrifugation process steps, performed using TAP Biosystems’ CompacT SelecT automated cell culture platform, upon the culture of a human induced pluripotent stem cell (hiPSC) line (VAX001024c07) were compared. This study, has demonstrated that comparable morphologies and cell diameters were observed in hiPSCs cultured using either manual or automated process steps. However, non-centrifugation hiPSC populations exhibited greater cell yields, greater aggregate rates, increased pluripotency marker expression, and decreased differentiation marker expression compared to centrifugation hiPSCs. A trend for decreased variability in cell yield was also observed after the utilisation of the automated process step. This study also highlights the detrimental effect of the cryopreservation and thawing processes upon the growth and characteristics of hiPSC cultures, and demonstrates that automated hiPSC manufacturing protocols can be successfully transferred between independent laboratories.

## Introduction

The reprogramming of adult somatic cells into pluripotent stem cells, known as induced pluripotent stem cells (iPSCs), was first achieved by Takahashi and Yamanaka [1] through the overexpression of Oct3/4, Sox2, Klf4, and c-Myc transcription factors. If cell therapies derived from Human iPSCs (hiPSCs) are to gain adoption in healthcare, consistent and scalable manufacturing processes will be essential. The automation of cell culture holds significant promise for the improvement of quality control, scalability, reproducibility and economics of the process [2, 3].

**Fig. 1:**
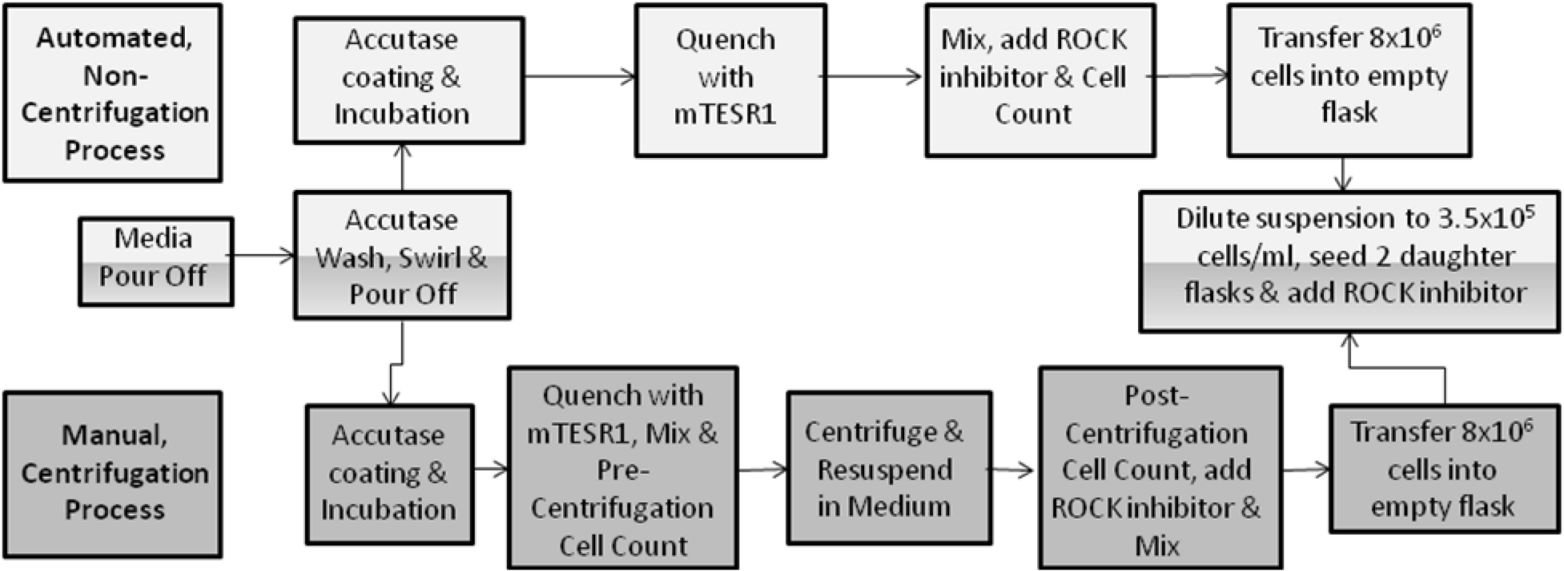
Process diagram illustrating the differences between the CompacT SelecT manual (centrifugation) and automated (non-centrifugation) hiPSC culture process steps

In this study, an automated culture process with an incorporated manual centrifugation process step, hence known as the ‘centrifugation’ process, was compared to a validated and fully automated culture process, which included an alternative ‘non-centrifugation’ process step resulting in residual dissociation agent remaining within the culture, hence known as the ‘non-centrifugation’ process. This allowed for the direct comparison of the effect of a manual centrifugation and an automated non-centrifugation process step upon hiPSC growth and characteristics by minimising the variability associated with fully manual culture processes. The differences in process steps between these processes are illustrated in Fig. 1.

**Fig. 2:**
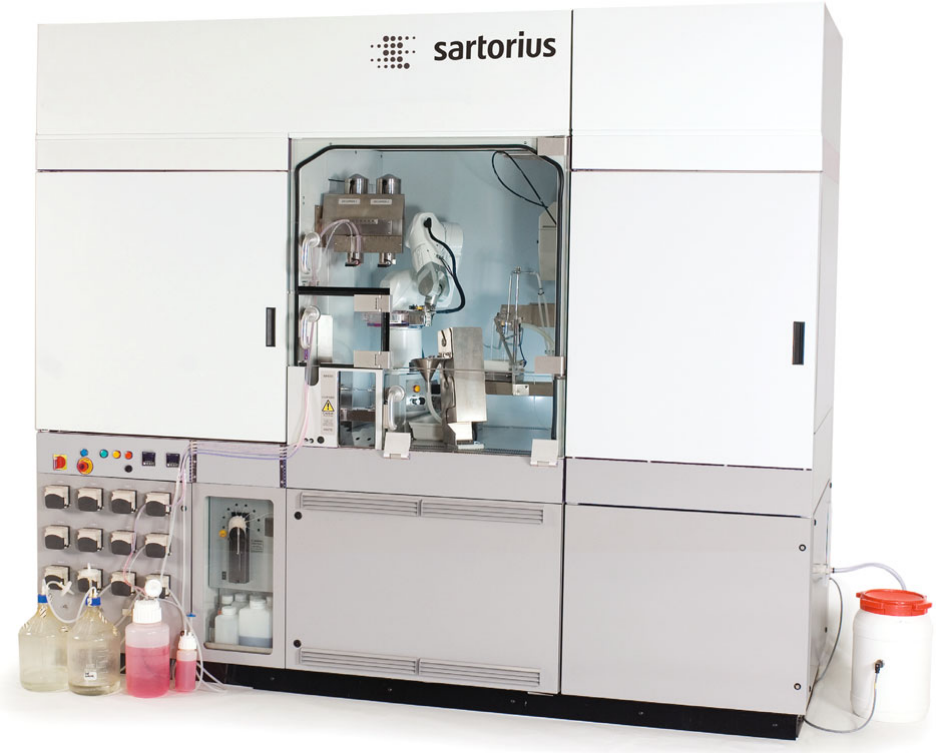
TAP Biosystems’ CompacT SelecT automated cell culture platform (TAP Biosystems, Part of the Sartorius Stedim Biotech Group, Royston, UK)

The CompacT SelecT automated cell culture platform (TAP Biosystems, Royston, UK) (Fig. 2); which utilises an incubator carousel to store cell culture flasks, multiple peristaltic pumps to dispense cell culture reagents, and a robotic arm to replicate many of the process steps involved in manual cell culture; was utilised in this study. This platform also has an automated cell counter incorporated; which utilises Trypan blue exclusion and automated imaging software to determine viable cell density, viability, and aggregate rate. The CompacT SelecT has previously been validated for the culture of hMSCs, hESCs and hiPSCs as aggregates [4–6]. Automated hiPSC culture protocols were transferred from I-Stem (Évry, France) to the Centre for Biological Engineering (Loughborough University, UK). However, these protocols required adaptation due to differences in the capabilities of the automated platforms located at each site. New protocols were generated to allow for the seeding and passage of a single T175 flask, and daily microscopy was utilised to determine culture confluency. The fully automated, non-centrifugation hiPSC culture protocol was also adapted to incorporate a centrifugation process step. This adapted protocol was utilised in the manual, centrifugation process arm of the experiment. By undertaking a process transfer between laboratories, product and process comparability between sites can be demonstrated, which can be crucial to achieving regulatory approval for biological products [7].

The recommended passage procedure for the VAX001024c07 hiPSC line involves the dissociation of the cells into a single cell suspension and their re-seeding onto Matrigel-coated tissue culture plastic. It has been identified that the dissociation of pluripotent stem cells into a single cell suspension can lead to apoptosis [8, 9]. However, the maintenance of pluripotent stem cell morphology over ten passages despite dissociation into single cells has been demonstrated [10]. Rho-associated protein kinase (ROCK) inhibitor has been shown to allow for the survival and maintenance of pluripotency of pluripotent stem cells after dissociation [11], as well as improved viable hiPSC recovery after cryopreservation [12, 13]. In this study, ROCK inhibitor was added to the culture during the initial seeding, passage and cryopreservation processes, as well as on Day 1 after initial seeding or passage.

Therefore, the aim of this study was to measure and establish the comparability between alternative process steps for the culture of the hiPSCs. This investigation examined the effects of manual and automated process steps upon the morphology, cell diameter, viable cell yield, viability, aggregate rate and pluripotency marker expression of the VAX001024c07 hiPSC line.

## Materials and methods

### hiPSC culture

#### Creation of a hiPSC working bank

The VAX001024c07 hiPSC line was generated at I-Stem by transducing human myoblasts with the four Yamanaka factors (OSKM) using amphotropic retroviruses and adapting the resulting hiPSCs to single cell culture, as described by Massouridès et al. [14]. Before transfer to Loughborough University, these cells had previously undergone 22 passages in the presence of feeder cells, and 9 passages in feeder-free conditions. These cells were then manually expanded, following the process steps and parameters of the automated ‘non-centrifugation’ culture process as closely as possible, to generate a working cell bank (‘Baseline hiPSCs’).

#### hiPSC centrifugation culture method

**Fig. 3:**
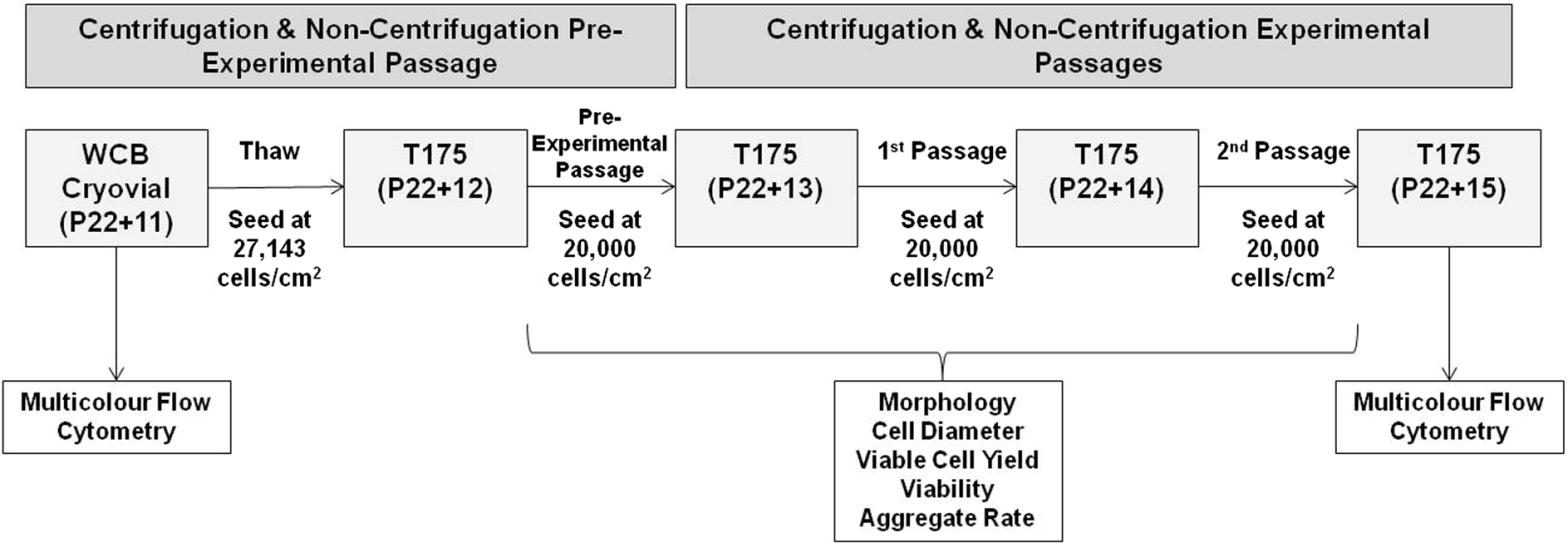
Process diagram describing the pre-experimental and experimental centrifugation and non-centrifugation passages. *WCB* working cell bank

Before any automated protocol was initiated on the CompacT SelecT platform (TAP Biosystems), the machine was prepared for use by loading sufficient consumables and reagents, and by performing calibrating and priming steps to ensure that the required volumes of each reagent is dispensed. As a result of test experimental runs performed during the process transfer between sites, it was determined that an additional passage prior to the experimental passages, or ‘pre-experimental’ passage, and an increased initial seeding density were required to mitigate the detrimental effects of cryopreservation upon hiPSC recovery. Therefore, these steps were incorporated into both the centrifugation and non-centrifugation experimental arms. An overview of the experimental workflow is illustrated in Fig. 3.

For each of the four centrifugation experimental runs, Baseline hiPSCs were thawed, suspended in pre-warmed mTeSR1 medium (StemCell Technologies, Vancouver, Canada), centrifuged at 276 RCF for 5 min, the supernatant aspirated, the cell pellet resuspended in mTeSR1 medium with ROCK inhibitor (10 µM) (Y-27632, StemCell Technologies), and the suspension transferred into a 50 ml centrifuge tube which was then placed in the static holder of the CompacT SelecT before an automated seeding protocol was performed. During this protocol, the cells were mixed, a cell count was performed, the cells were diluted, and 4.75 × 10^6^ cells (2.7143 × 10^4^ cells/cm^2^) were transferred into a new Matrigel-coated barcoded T175 flask (P22 + 12). Matrigel™ (BD Biosciences, San Jose, USA) was diluted with Knockout™ DMEM (322.5 μg/ml) (Life Technologies, Thermo Fisher Scientific, Waltham, USA). A medium exchange with mTESR1 10 μM ROCK inhibitor solution was performed 4 h after seeding, once the viable cells had adhered to the flask, to remove dead or non-adherent cells; as well as 24 h after initial seeding. Subsequently, every 24 h, confluency was examined using microscopy and a medium exchange with mTeSR1 was performed.

To passage these cells, after approximately 7 days and once 80 % confluent, the cells were pre-treated with 10 μM ROCK inhibitor solution for 1 h and an automated pre-centrifugation passage protocol was performed to dissociate the cells with Accutase™ (StemCell Technologies); agitate any non-dissociated cells; quench with mTeSR1; and obtain cell count, viability, aggregation and cell diameter data. The mother flask containing the dissociated cells was then “outfeeded”, which refers to the temporary ejection of a flask from the platform and allows the flask to be re-imported and recognised by the CompacT SelecT software. The cell suspension was then centrifuged, the cells were resuspended in fresh mTeSR1, thoroughly mixed, and reintroduced into the mother T175 flask which was imported back into the CompacT SelecT. Next, an automated post-centrifugation protocol was utilised to perform a cell count, add 3 ml of 10 μM ROCK inhibitor solution, isolate 8 × 10^6^ cells, dilute the isolated cells, seed the appropriate number of Matrigel-coated daughter flasks with 3.5 × 10^6^ cells, and add additional mTeSR1 and ROCK inhibitor solution to each daughter flask. Daily medium exchanges were performed after each passage, with ROCK inhibitor added on Day 1. The following formula was utilised to determine the cumulative population doublings (CPDs) for each hiPSC experimental run:

$${\text{CPDs}} = [\text{Time of Final Cell Count }(\text{Days}) -\text{Time of Seeding } (\text{Days})]/\text{Population Doubling Time }(\text{Days})$$

For each passage, identical protocols were utilised. However, during the pre-experimental passage, each T175 flask was passaged into a single T175 daughter flask, whereas in later passages two daughter flasks were seeded from each mother flask. This low flask expansion rate was utilised, in accordance with the I-Stem manufacturing protocols, to allow for a sufficient number of passages to facilitate hiPSC recovery post-thaw, and to allow for multiple batches to be performed without exceeding the capacity of the CompacT SelecT incubator. It must be noted that centrifugation cell count data could not be collected during the 2nd passage of the fourth batch due to a malfunction of the Cedex Automated Cell Counter, which is integrated in the CompacT SelecT platform. After three passages, and once 80 % confluent, the four T175 flasks generated per batch were pre-treated with 10 μM ROCK inhibitor solution, harvested, the cells pooled, counted, resuspended in Cryostor^®^ CS-10 freezing medium, and cryopreserved.

#### hiPSC non-centrifugation culture method

For each of the four non-centrifugation experimental runs, similar cell revival and resuspension processes to those described in the hiPSC centrifugation culture method were utilised during seeding of the mother flask, as well as the inclusion of a ‘pre-experimental’ passage and an increased initial seeding density. Furthermore, an identical automated seeding protocol and medium exchange frequency was utilised.

Once 80 % confluent, the cells were pre-treated with 10 μM ROCK inhibitor solution for 1 h and an automated non-centrifugation protocol was performed, in which residual dissociation agent was not removed and was carried over throughout culture. During this protocol, the cells were washed with Accutase, incubated with Accutase for 10 min at 37 °C, agitated to dissociate any adherent cells, and quenched with mTeSR1 medium with 10 μM ROCK inhibitor solution. A cell count was also performed, 8 × 10^6^ cells were isolated, isolated cells were diluted, and the appropriate number of new barcoded Matrigel-coated daughter flask were seeded with 3.5 × 10^6^ cells.

For each passage, identical pre-treatment, and non-centrifugation protocols were utilised. Similarly to the centrifugation arm, after the pre-experimental passage, the number of daughter flasks seeded from each mother flask increased to two. Due to a malfunction of the Cedex Automated Cell Counter, non-centrifugation granddaughter flask cell count data could not be collected for the fourth batch. After three passages, identical harvesting and downstream processing steps to those described for the centrifugation arm were utilised.

### Pluripotency marker expression

Baseline (P22 + 11), centrifugation (P22 + 15) and non-centrifugation (P22 + 15) hiPSCs were prepared for multicolour flow cytometry following the manufacturer’s instructions (BD Stemflow, BD Biosciences) after a cell count was performed. To determine the immunophenotype of each hiPSC population, multicolour flow cytometry was performed using antibodies for two markers which are commonly used to identify undifferentiated pluripotent stem cells, specifically Stage-Specific Embryonic Antigen-3 (SSEA-3) and Tumour-Related Antigen-1-81 (TRA-1-81), and for one marker which has been identified as a negative marker of pluripotent cells, specifically SSEA-1 [15, 16].

Briefly, after fixation, the cells were added to flow cytometry tubes (BD Biosciences) containing either; one of the FITC SSEA-1, PE SSEA-3 and Alexa Fluor 647 TRA-1-81 antibodies, or one of the FITC, PE and Alexa Fluor 647 isotype controls. Replicates of each of the specific stain and isotype control tubes were generated, and all tubes incubated in the dark on ice for 30 min and washed with stain buffer. Control beads were also prepared with the appropriate antibodies. Prior to analysing hiPSC samples on the BD FACSCanto II (BD Biosciences) using the FACSDiva software version 6.1.3, unstained negative beads were analysed. A gate was then set around the singlet bead population on the FSC vs SSC plot; and the FITC, PE, and Alexa Fluor 647 stained control beads were analysed to ensure that the positive populations fitted on the FSC and SSC scales, and to calculate compensation. Unstained cells were then analysed, and a gate was set around the main cell population on the FSC vs SSC plot. Next, the isotype controls were analysed, followed by the hiPSC samples. For analysis, the flow cytometry data was exported in FCS 3 format and analysed using FlowJo software v10. Scatter plots for the isotype controls are presented in Fig. 13a–c in Appendix.

### Statistical analyses

Viable cell density, viable cell yield, population doubling time, viability, cell diameter, and aggregate rate data for pre-centrifugation, post-centrifugation and non-centrifugation hiPSCs across all passages was analysed using two-way analysis of variance (ANOVA) multiparameter analyses, through the IBM SPSS statistical software, to determine significant differences. Furthermore, one-way ANOVAs were utilised to assess the significance of differences in viable cell density, viable cell yield, and viability of pre-centrifugation, post-centrifugation and non-centrifugation of hiPSCs within the 2nd passage. One-way ANOVAs were also used to assess the significance of differences between the standard deviations (SD) of the viable cell densities, viable cell yields, and viabilities of pre-centrifugation, post-centrifugation, and non-centrifugation of hiPSCs, in each of the four batches, from the second passage. The cut-off value for statistical significance (*p*) was set at 0.05.

## Results

### Morphology

**Fig. 4:**
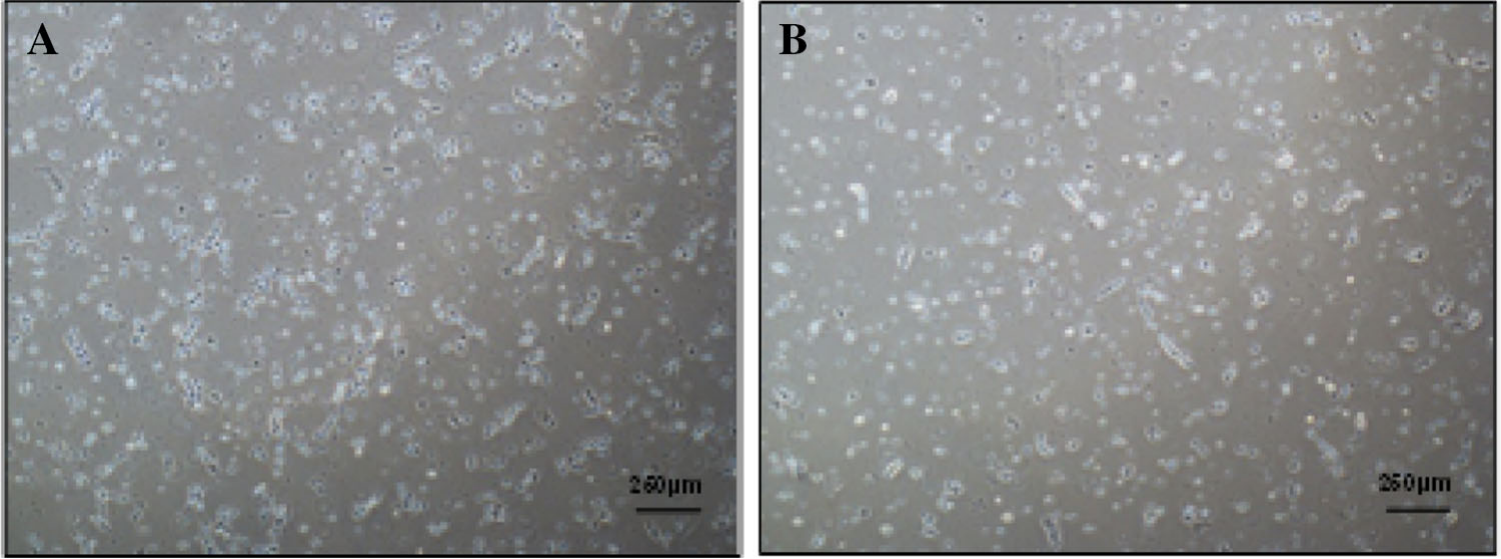
hiPSC batch 3 post-centrifugation (Ce) (**a**) and non-centrifugation (NC) (**b**) Day 1 Morphology

**Fig. 5:**
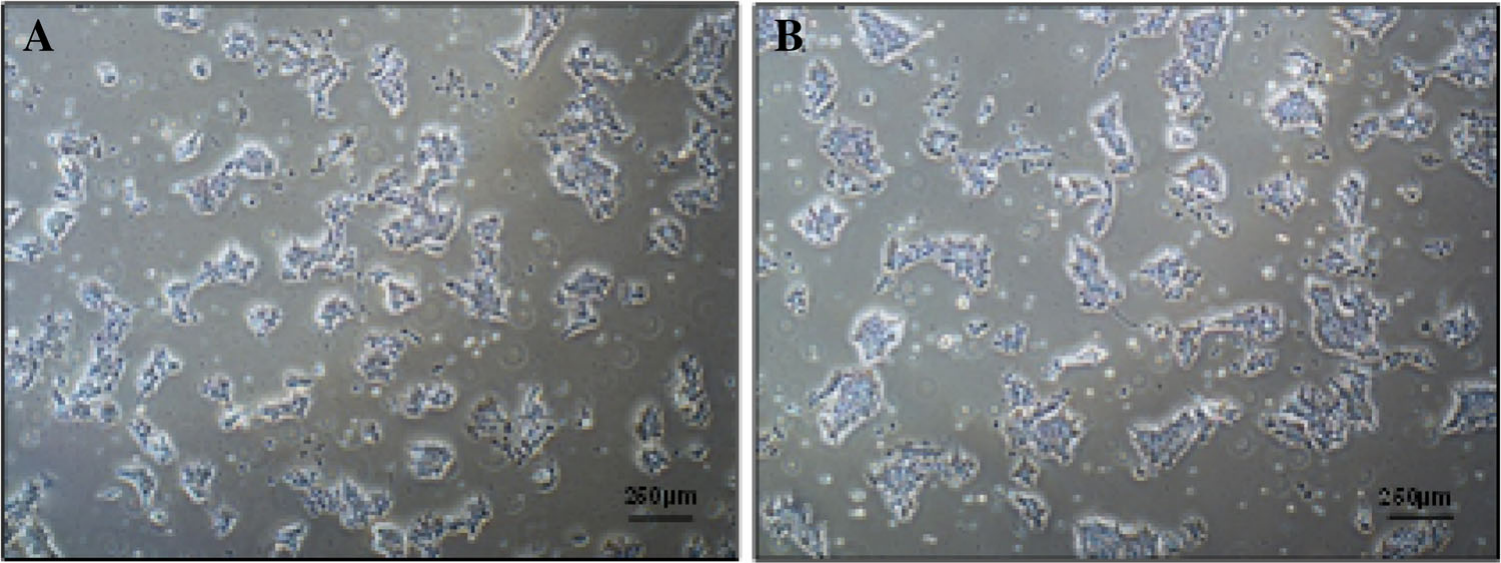
hiPSC batch 3 post-centrifugation (Ce) (**a**) and non-centrifugation (NC) (**b**) Day 4 Morphology

After visual examination, it was determined that single cells with multiple long, thin lamellipodia were generated early in culture (Days 0–1) (Fig. 4a, b), and that, after 48 h of culture, hiPSCs began to form small colonies with a rounded morphology, often with few spontaneously differentiated cells at their periphery (Fig. 5a, b). Exemplar images of small hiPSC colonies, cultured using the centrifugation and non-centrifugation protocols, visualised under a higher magnification are presented in Fig. 14 in Appendix. Cell populations cultured using centrifugation and non-centrifugation process steps observed similar morphologies both early (Days 0–1) and later (≥Day 2) in culture. However, it appeared that hiPSCs cultured using the non-centrifugation process step formed small colonies more rapidly than those cultured using the centrifugation process step, despite the addition of ROCK inhibitor early in culture (Days 0–1).

### Cell diameter

**Fig. 6:**
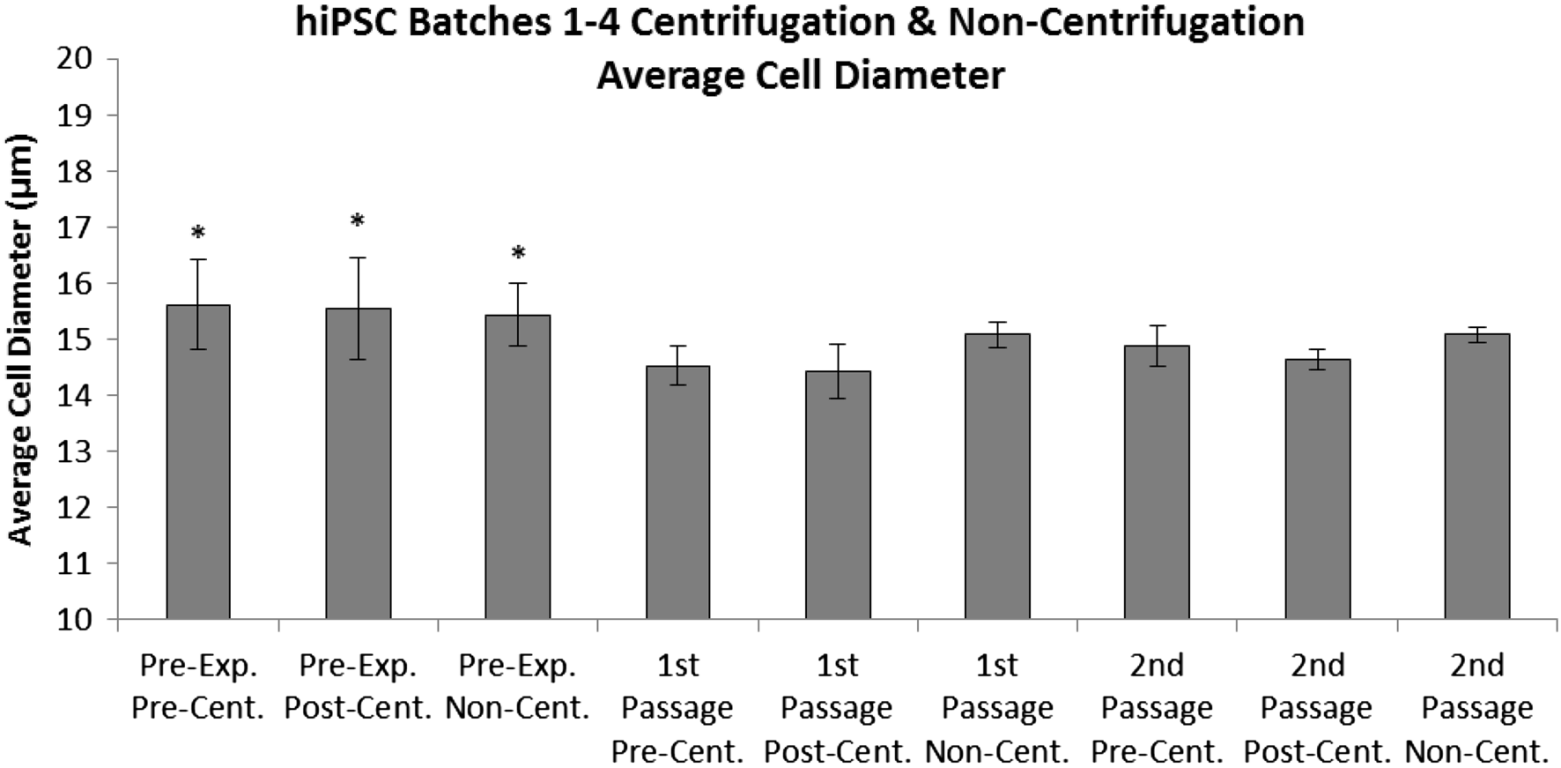
The average cell diameter of pre-centrifugation, post-centrifugation and non-centrifugation hiPSCs from all four batches and over the pre-experimental [replicates (*n*) = 4], 1st [replicates (*n*) = 4], and 2nd passages [replicates (*n*) = 6]. Standard deviations are plotted as *error bars*. *Asterisk* (*) denotes significance over 1st and 2nd passages (*p* = 0.05)

After comparing the cell diameter of hiPSC populations across all passages, it was determined that cells from the pre-experimental passage (P22 + 12/P34), cultured after cryopreservation, were significantly larger than cells from the 1st (P22 + 13/P35) and 2nd (P22 + 14/P36) passages regardless of the process step utilised (*p* < 0.001) (Fig. 6). No significant difference in cell diameter was observed between Pre-, post- and non-centrifugation hiPSCs populations across all passages, demonstrating that neither the automated nor the manual process steps significantly influenced cell size.

### Pluripotency marker expression

**Fig. 7:**
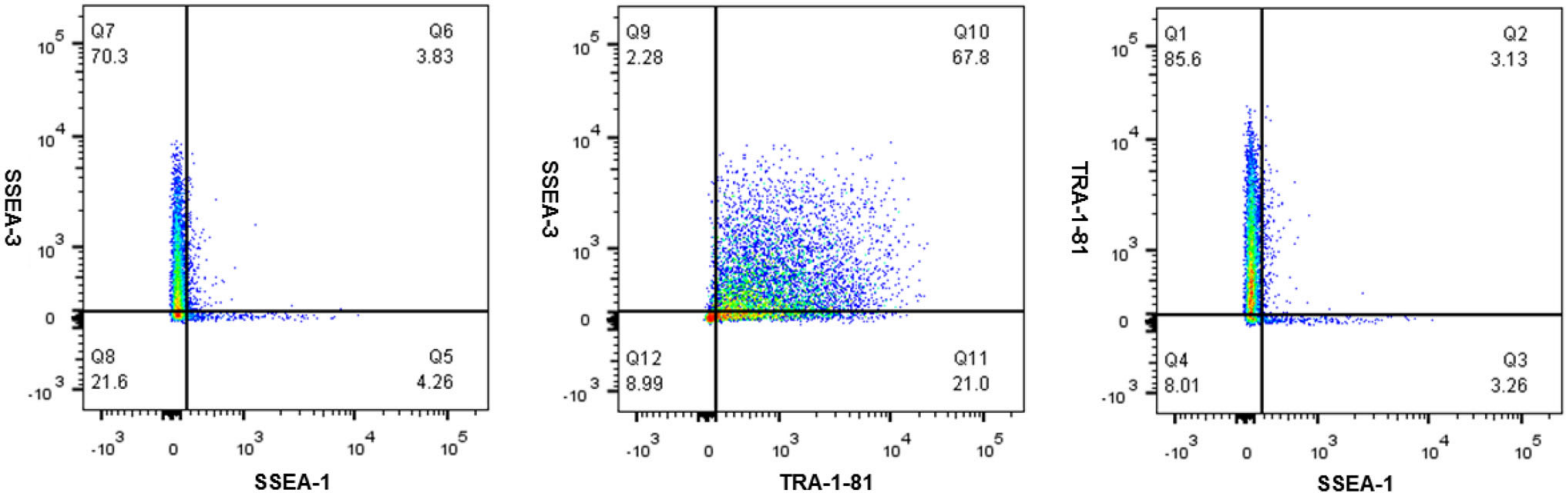
Scatter plots demonstrating multicolour flow cytometric analysis of pluripotency and differentiation marker co-expression of baseline hiPSCs from the working cell bank (P22 + 11)

**Fig. 8:**
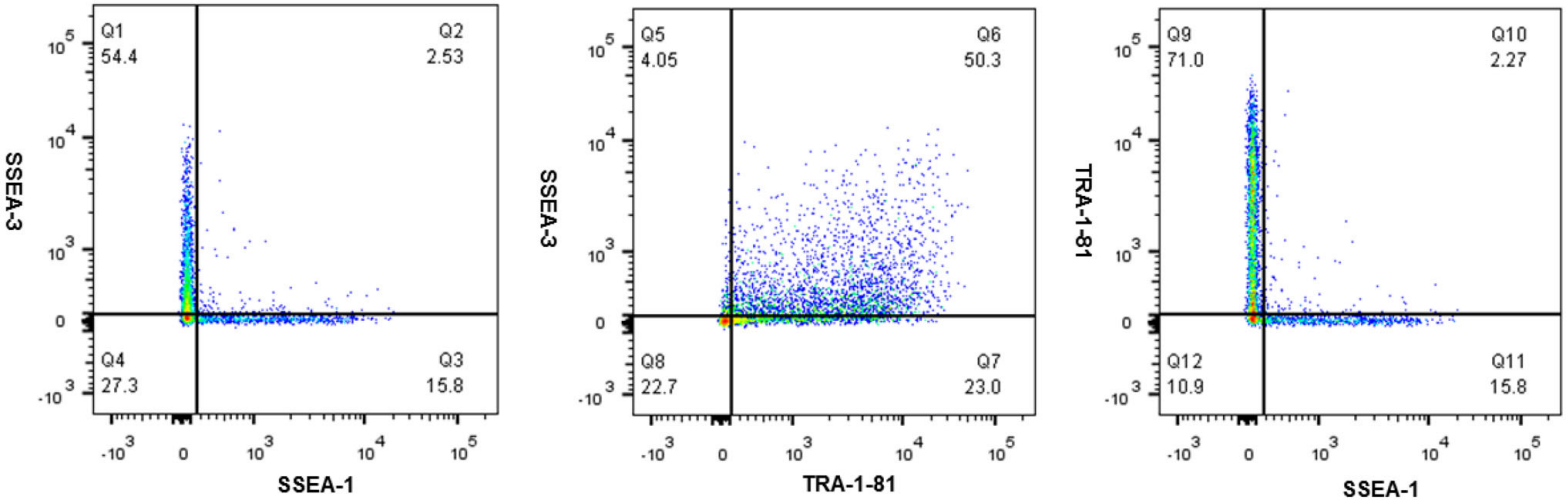
Scatter plots demonstrating multicolour flow cytometric analysis of pluripotency and differentiation marker co-expression of centrifugation hiPSCs (P22 + 15)

**Fig. 9:**
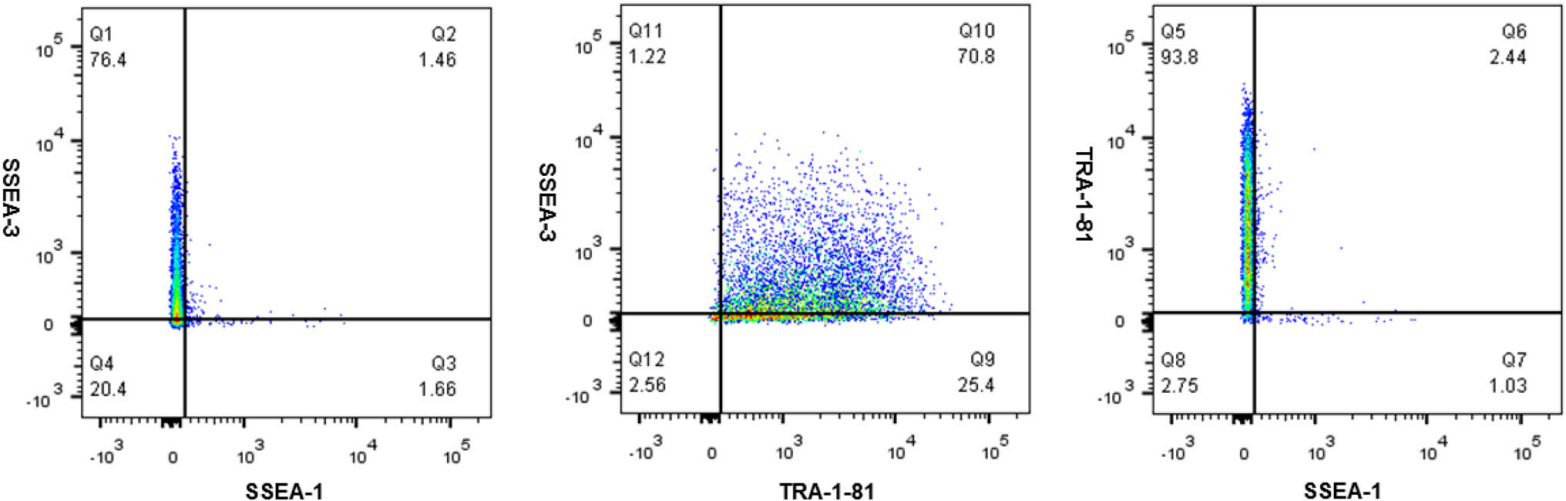
Scatter plots demonstrating multicolour flow cytometric analysis of pluripotency and differentiation marker co-expression of non-centrifugation hiPSCs (P22 + 15)

Human iPSCs have been previously demonstrated to express the pluripotency markers TRA-1-81 and SSEA-3, and to lack the expression of the SSEA-1 differentiation marker [17]. In this study, single cell analysis of the immunophenotype of baseline (P22 + 11), centrifugation (P22 + 15) and non-centrifugation (P22 + 15) of hiPSCs revealed that the majority of cells in each population co-expressed SSEA-3 and TRA-1-81 (50.3–70.8 %) (Figs. 7, 8, 9), and that the expression of SSEA-1 was low in all hiPSC populations (<20 %). These findings indicate that each of the baseline, centrifugation and non-centrifugation hiPSC populations contained predominantly pluripotent cells.

However, after comparing the immunophenotype of hiPSC populations from each condition, it was observed that centrifugation of hiPSC populations exhibited a reduced pluripotency marker expression, and an increased differentiation marker expression, compared to both baseline and non-centrifugation of hiPSC populations.

### Viable cell yield

**Fig. 10:**
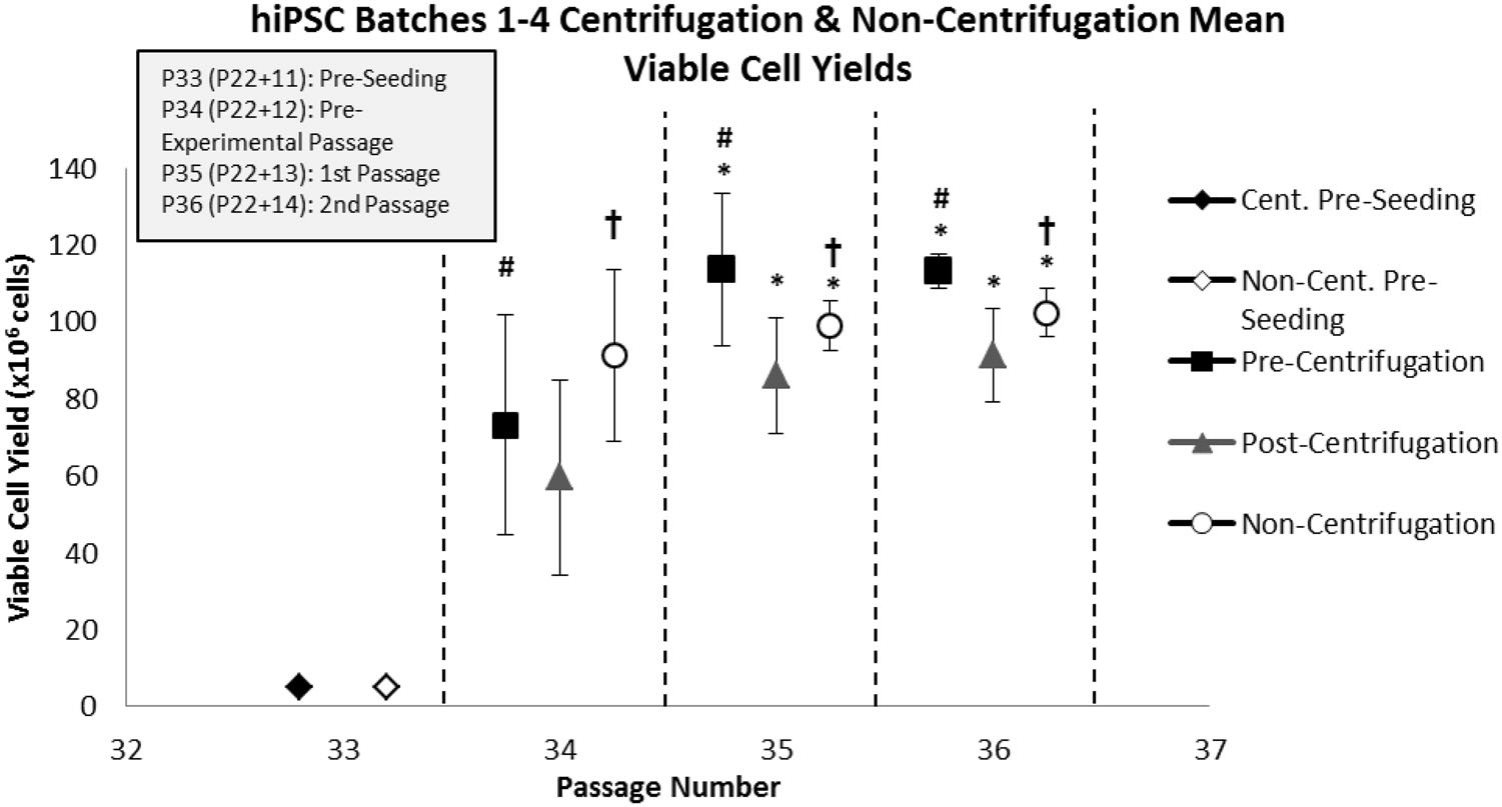
The average pre-centrifugation, post-centrifugation and non-centrifugation viable hiPSC yield per flask over the pre-experimental (P34/P22 + 12) [replicates (*n*) = 4], 1st (P35/P22 + 13) [replicates (*n*) = 4], and 2nd (P36/P22 + 14) [replicates (*n*) = 6] passages, and across four batches. Standard deviations are plotted as *error bars*. *Asterisk* (*) denotes significance over pre-experimental passage (*p* = 0.05). *Number sign* (#) denotes significance of pre-centrifugation hiPSCs over post-centrifugation hiPSCs over all passages (*p* = 0.05). *Dagger symbol* (†) denotes significance of non-centrifugation hiPSCs over post-centrifugation hiPSCs over all passages (*p* = 0.05)

As illustrated in Fig. 10, the viable cell yields of hiPSC populations in the 1st (P35/P22 + 13) (*p* = 0.003) and 2nd (P36/P22 + 14) (*p* < 0.001) passages were significantly greater than those in the pre-experimental (P34/P22 + 12) passage regardless of the process step utilised. It was also observed that pre-centrifugation hiPSC populations demonstrated significantly greater viable cell yields compared to post-centrifugation hiPSC populations over all passages (*p* = 0.007). Furthermore, non-centrifugation hiPSC populations exhibited significantly greater viable cell yields compared to post-centrifugation hiPSC populations over all passages (*p* = 0.028). Although no significant difference in viable cell yield between pre-centrifugation and non-centrifugation hiPSC populations was observed, given the reduction in viable cell yield after the centrifugation process, it is apparent that a greater viable cell yield is achievable when the non-centrifugation process step is utilised. Mean cumulative population doublings (CPDs) of 17.61 and 17.72 were achieved after culture over three passages utilising the centrifugation and non-centrifugation process steps, respectively.

By comparing the standard deviations of viable cell yields for pre-, post- and non-centrifugation hiPSC populations, it was determined that no significant difference in the variability in viable cell yield was observed between process steps in the 2nd passage. However, a non-significant trend for a lower variability in viable cell yield in non-centrifugation samples compared to post-centrifugation samples, may exist.

### Viability

**Fig. 11:**
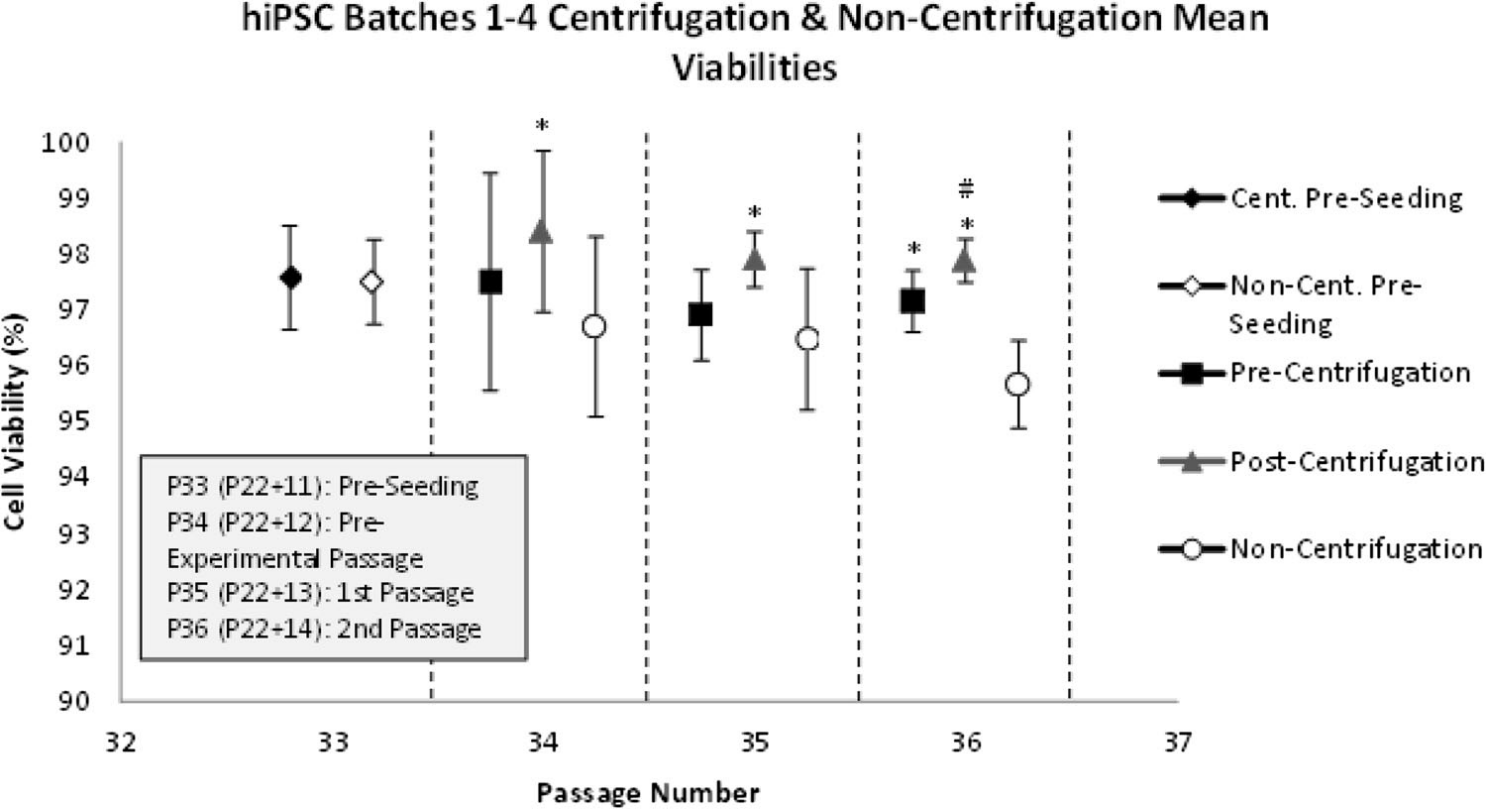
The mean viability of pre-centrifugation, post-centrifugation and non-centrifugation hiPSC samples over the pre-experimental (P34/P22 + 12) [replicates (*n*) = 4], 1st (P35/P22 + 13) [replicates (*n*) = 4], and 2nd (P36/P22 + 14) [replicates (*n*) = 6] passages, and across four batches. Standard deviations are plotted as *error* *bars*. *Asterisk* (*) denotes significance over non-centrifugation hiPSCs (*p* = 0.05). *Number sign* (#) denotes significance over pre-centrifugation hiPSCs in the 2nd passage (*p* = 0.05)

No significant difference in hiPSC viability was observed between the pre-experimental (P34/P22 + 12), 1st (P35/P22 + 13) or 2nd (P36/P22 + 14) passages (Fig. 11). It was also demonstrated that the viability of non-centrifugation hiPSC samples was significantly lower than that of post-centrifugation samples across all passages (*p* < 0.001), and was significantly lower than that of pre-centrifugation samples in the 2nd passage (*p* = 0.005). Furthermore, significantly greater hiPSC viability was observed in post-centrifugation samples compared to pre-centrifugation samples in the 2nd passage (*p* = 0.045). Finally, when the standard deviations of hiPSC viabilities were compared, no significant difference in the variability in hiPSC viability between process steps was observed, although non-significant trends for greater variability in non-centrifugation hiPSC samples, as well as those from the pre-experimental passage.

### Average aggregate rate

**Fig. 12:**
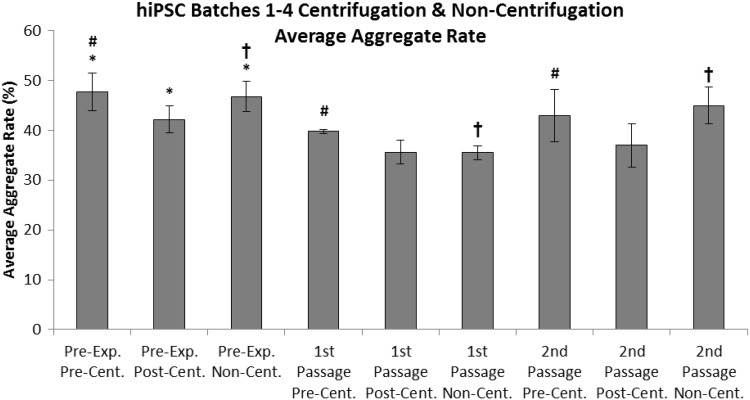
Average aggregate rate from pre-, post- and non-centrifugation hiPSC counts from all four batches over the pre-experimental (P34/P22 + 12) [replicates (*n*) = 4], 1st (P35/P22 + 13) [replicates (*n*) = 4], and 2nd (P36/P22 + 14) [replicates (*n*) = 6] passages. Standard deviations are plotted as *error bars*. *Asterisk* (*) denotes significance of pre-experimental passage over 1st and 2nd passages (*p* = 0.05). *Number sign* (#) denotes significance of pre-centrifugation hiPSCs over post-centrifugation hiPSCs (*p* = 0.05). *Dagger symbol* (†) denotes significance of non-centrifugation hiPSCs over post-centrifugation hiPSCs (*p* = 0.05)

**Fig. 13:**
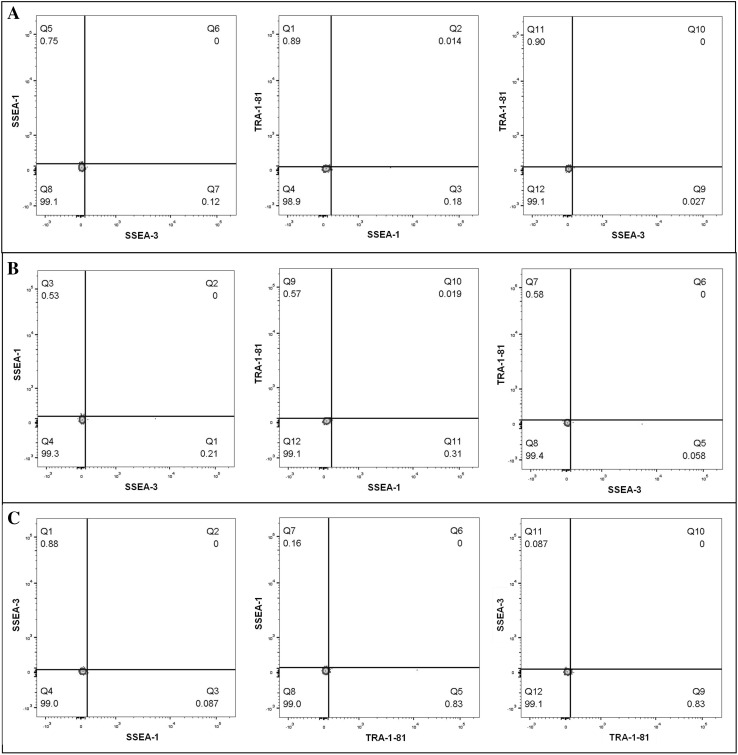
**a** *Top*, **b** *middle* and **c** *bottom*. Baseline (**a**), centrifugation (**b**) and non-centrifugation (**c**) hiPSC isotype control multicolour flow cytometry scatter plots

**Fig. 14:**
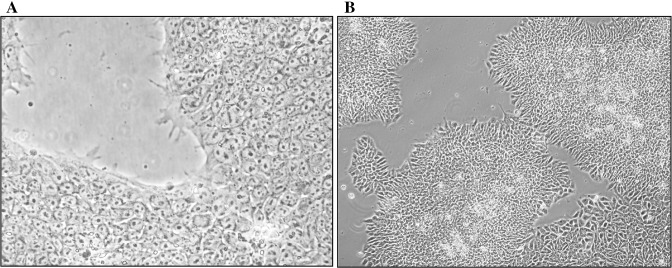
**a** *Left* and **b** *right.* Exemplar images of a non-centrifugation (**a**) and post-centrifugation (**b**) hiPSC culture visualised under high magnification

By comparing the average aggregate rate of hiPSC populations from all four batches, over three passages, it was determined that the aggregate rate of populations in the pre-experimental passage (P34/P22 + 12) were significantly higher than those from the 1st (P35/P22 + 13) (*p* = 0.001) and 2nd passages (P36/P22 + 14) (*p* = 0.017), regardless of the process step utilised (Fig. 12). It was also determined that significantly greater aggregation was observed in pre-centrifugation hiPSC samples compared to post-centrifugation samples over all passages (*p* = 0.001). Furthermore, it was determined that aggregate rate was significantly higher in non-centrifugation hiPSC samples compared to post-centrifugation samples, across all passages (*p* < 0.001).

## Discussion and conclusions

The VAX001024c07 hiPSC line utilised in this study was selected as it has previously been adapted to single cell, monolayer culture and has been demonstrated by I-Stem to be amenable to culture using the CompacT SelecT automated platform. This study has demonstrated that comparability in cell morphology and diameter was observed between hiPSCs cultured using manual and automated process steps. Furthermore, no significant difference in cell diameter was observed between process steps. However, non-centrifugation hiPSC populations exhibited greater cell yields, greater aggregate rates, and lower cell viabilities compared to centrifugation hiPSCs. The decrease in viable cell yield after the manual, centrifugation process step may indicate that further optimisation of this step is required for the culture of hiPSCs. In particular, optimisation of the RCF utilised, and standardisation of the supernatant aspiration step, may be required. Furthermore, a trend for decreased variability in viable cell yield was also observed after the utilisation of the non-centrifugation process step, which suggests that the automated process step allowed for more consistent viable hiPSC yields to be achieved.

The greater aggregate rates observed in non-centrifugation populations, and the decrease in aggregate rate identified after the performance of the centrifugation process step, indicate that the automated process step does not allow for comparable aggregate dissipation to that of the manual process step; and that the process of centrifugation reduces the amount of cell aggregation. However, the incorporated mixing steps performed after the non-centrifugation cell count may partly mitigate this increased aggregation. These findings suggest that the centrifugation process step may be beneficial for the generation of a single cell suspension, which is favourable for seeding the VAX001024c07 hiPSC line and cell enumeration using the Cedex automated cell counter.

The lower cell viabilities observed in non-centrifugation populations may suggest that the automated process step negatively impacted the viability of hiPSC populations, which may be linked to the residual dissociation agent carryover. However, the difference between the mean viabilities for post-centrifugation and non-centrifugation samples was ≤2.2 %, which is below the maximum intra-sample variability in cell viability for the Cedex automated cell counter (±3 %) [18], with the viability of non-centrifugation populations remaining above 94 % throughout the experiment. The increase in hiPSC viability observed after the centrifugation process step may be associated with the removal of debris and non-viable cells.

All hiPSC populations in this study demonstrated ≤10 % SSEA-1 expression and ≥70 % TRA-1-81 expression, therefore, meeting the I-Stem criteria for expression of these markers are required during manufacturing runs. However, only baseline and non-centrifugation populations met the ≥70 % SSEA-3 expression criteria. Furthermore, although the majority of hiPSCs in each population co-expressed SSEA-3 and TRA-1-81, only non-centrifugation hiPSC populations were shown to meet the I-Stem criteria of ≥70 % co-expression of SSEA-3 and TRA-1-81 pluripotency markers. Therefore, non-centrifugation populations exhibited increased pluripotency marker expression and decreased differentiation marker expression compared to centrifugation hiPSCs, which may indicate that the automated process step was favourable for the maintenance of pluripotency in hiPSC cultures. Previous studies have also identified a reduction in pluripotency marker expression after centrifugation [3, 19].

This study also highlights the detrimental effect of the cryopreservation and thawing processes upon hiPSC populations, with these cells exhibiting larger cell diameters, lower viable cell yields, greater population doubling times, and greater aggregate rates in the first passage after cryopreservation. It is, therefore, apparent that these cells require one passage after cryopreservation to recover their typical characteristics. However, the cryopreservation and thawing processes did not impact hiPSC viability, which may be due to the performance of daily medium exchanges and the resultant removal of apoptotic cells.

Finally, this study demonstrates that an automated hiPSC manufacturing process transfer between independent laboratories can be successfully completed, allowing for the generation of high hiPSC yields which predominantly co-express the SSEA-3 and TRA-1-81 pluripotency markers. However, as a single hiPSC line was examined in this study, further research is required to determine the way in which other hiPSC lines derived from different donors would respond to the utilisation of alternative process steps. Furthermore, as three automated passages were performed for each centrifugation and non-centrifugation batch, the comparability between process steps over extended culture cannot be determined from this study.

## Appendix

See Figs. 13 and 14.

## Abbreviations

ANOVA: Analysis of variance
CLA: Cell line authentication
CPDs: Cumulative population doublings
FSC: Forward scatter
HSD: Honest significant difference
hiPSC: Human induced pluripotent stem cell
iPSC: Induced pluripotent stem cell
RCF: Relative centrifugal force
ROCK inhibitor: Rho-associated protein kinase inhibitor
SD: Standard deviation
SSC: Side scatter
SSEA: Stage-specific embryonic antigen
TRA: Tumour-related antigen
WCB: Working cell bank
